# Identifying Metabolic Inhibitors to Reduce Bacterial Persistence

**DOI:** 10.3389/fmicb.2020.00472

**Published:** 2020-03-27

**Authors:** Sayed Golam Mohiuddin, Thuy Hoang, Adesola Saba, Prashant Karki, Mehmet A. Orman

**Affiliations:** Department of Chemical and Biomolecular Engineering, University of Houston, Houston, TX, United States

**Keywords:** persister cells, stationary-phase metabolism, metabolic inhibitors, drug screening, viable but non-culturable cells

## Abstract

Bacterial persisters are rare phenotypic variants that are temporarily tolerant to high concentrations of antibiotics. We have previously discovered that stationary-phase-cell subpopulations exhibiting high redox activities were less capable of producing proteins and resuming growth upon their dilution into fresh media. The redox activities of these cells were maintained by endogenous protein and RNA degradation, resulting in self-inflicted damage that transiently repressed the cellular functions targeted by antibiotics. Here, we showed that pretreatment of stationary-phase cells with an ATP synthase inhibitor, chlorpromazine hydrochloride (CPZ), significantly reduced stationary-phase-redox activities and protein degradation, and yielded cells that were more susceptible to cell death when exposed to antibiotics in fresh media. Leveraging this knowledge, we developed an assay integrating a degradable fluorescent protein system and a small library, containing FDA-approved drugs and antibiotics, to detect medically relevant drugs that potentially target persister metabolism. We identified a subset of chemical inhibitors, including polymyxin B, poly-L-lysine and phenothiazine anti-psychotic drugs, that were able to reduce the persistence phenotype in *Escherichia coli*. These chemical inhibitors also reduced *Pseudomonas aeruginosa* persistence, potentially verifying the existence of similar mechanisms in a medically relevant organism.

## Introduction

Conventional therapies for infectious diseases target the mechanisms that enable the rapid growth of bacterial cell populations. Although this can provide a clinical benefit, this benefit is usually short-lived for persistent and recurrent infections, and a large body of evidence suggests that small subpopulations of microbial cells invariably survive this initial selection pressure. One of the proposed mechanisms for this tolerance is via the establishment of a latent pool of persister cells (Van den Bergh et al., 2017). Persisters are an important health problem, because they are thought to underlie the propensity of recurrent infections to relapse (Lewis, 2007, 2010; Fauvart et al., 2011) and serve as a reservoir from which drug-resistant mutants can emerge (Van den Bergh et al., 2016; Levin-Reisman et al., 2017; Barrett et al., 2019; Windels et al., 2019). Persisters exhibit a diverse range of proliferative, metabolic, and transcriptional activities. Whereas there are some variants that can grow in the presence of antibiotics, these are very rare and often survive the drug treatments by bypassing the pathways targeted by the drugs (Wakamoto et al., 2013). By contrast, the most abundant persister variants do not grow in the presence of antibiotics and are largely formed before or during the antibiotic treatments (Balaban et al., 2004; Lewis, 2007, 2010, 2012). Elucidating the formation mechanisms of persister cells is of special interest; because, these cells are found among many bacterial species, are often multidrug tolerant, and their eradication is a huge challenge (Lewis, 2007, 2010; Van den Bergh et al., 2017).

Although persister cells are characterized by a lack of proliferation, they may still exist in a metabolic state, where energy is continually produced and consumed, without generating significant biomass (Radzikowski et al., 2016). In fact, persister cells formed during a nutrient shift were shown to produce more ATP (per mol of consumed carbon source) than the normal cells (Radzikowski et al., 2016). Persister cells are also known to metabolize certain carbon sources which make them susceptible to aminoglycosides (Allison et al., 2011; Orman and Brynildsen, 2013b; Orman et al., 2015). This susceptibility was conferred by increased aminoglycoside uptake due to the increased electron transport chain (ETC) activities and membrane potential, which is facilitated by catabolism of the carbon sources (Allison et al., 2011).

Although metabolic processes and persistence in bacteria are closely related (Amato et al., 2014; Prax and Bertram, 2014), the specific mechanisms that link these remain largely unknown. We previously showed that stationary-phase cells with high redox activities, maintained by endogenous protein and RNA degradation, were enriched with antibiotic-tolerant cells that couldn’t resume growth upon exposure to fresh nutrients (Orman and Brynildsen, 2015). We speculated that protein and RNA degradation not only provided energy to bacterial cells in a non-nutritive environment, but also produced self-inflicted damage that renders the cells less fit for rapid resumption of growth. Inhibiting stationary-phase-respiratory activities chemically (treatment with potassium cyanide or nitric oxide to suppress cellular respiration), environmentally (culturing under anaerobic conditions), or even genetically (genes encoding redox enzymes such as *ubiF*, *sucB*, *mdh*, *aceE*, *sdhC*, and *acnB*) reduced persister levels by preventing digestion of endogenous proteins and RNA, yielding cells that were more capable of translation and replication and thus susceptible to cell death when they were diluted into fresh media in the presence of antibiotics (Orman and Brynildsen, 2015, 2016). This reduction in persister levels was not found to be associated with the inhibition of RNA and protein synthesis or elimination of reactive oxygen species (ROS) (Orman and Brynildsen, 2015).

Our aim in this study is to detect medicinally relevant inhibitors that can reduce persistence by inhibiting bacterial cell metabolism. As effective sterilization methods for treating chronic and recurrent infections remain scarce, identifying novel targets, together with the inhibitors, is becoming an urgent priority to improve the therapies for these infections. Chlorpromazine, which is an FDA approved antidepressant drug that is effective, safe and listed as an essential medicine by the World Health Organization (WHO, 2019), was demonstrated to inhibit the catalytic complex of rotary nanomotor ATP synthase (F1-ATPase) in *E. coli* cells (Chazotte et al., 1982; Bullough et al., 1985). Here, we show that pretreatment of stationary-phase cells with chlorpromazine hydrochloride (CPZ) significantly reduces cellular redox activities, protein degradation and antibiotic-tolerant cell formation. Using a high-throughput screening approach and a small chemical library (Biolog Phenotype Arrays containing FDA-approved drugs and antibiotics), we further identify a subset of drugs that can reduce antibiotic-tolerant cells in Gram-negative bacteria by targeting their metabolism.

## Materials and Methods

### Bacterial Strains and Plasmids

*Escherichia coli* MG1655 wild-type (WT) and MO strains as well as pQE-80L plasmids harboring genes encoding degradable (SsrA-tagged) green fluorescent protein (GFP) were obtained from Dr. Mark P. Brynildsen at Princeton University (Supplementary Table S1). *Pseudomonas aeruginosa* PAO1 was a gift from Dr. Vincent Tam at the University of Houston (Supplementary Table S1). *E. coli* MO strain harbors a chromosomally integrated isopropyl β-D-1-thiogalactopyranoside (IPTG)-inducible *mCherry* expression cassette, which is used to monitor cell proliferation at single cell level (Orman and Brynildsen, 2013a, b, 2015). pQE-80L expression system has an IPTG-inducible synthetic *T5* promoter and a strong constitutive *LacI*^*q*^ promoter (with a point mutation) as a repressor, enabling us to tightly regulate the expression of SsrA-tagged GFP (Orman and Brynildsen, 2015). To directly measure protein degradation rates in stationary-phase cultures, we employed an assay using SsrA, a short peptide degradation tag with 11 amino acid residues that is linked to GFP to mark it for degradation by cellular proteases (Herman et al., 1998; Flynn et al., 2001; Spiers et al., 2002; Weichart et al., 2003; Choy et al., 2007). The overexpression of fluorescent proteins on *E. coli* persistence was shown to be insignificant (Orman and Brynildsen, 2013a, b, 2015, 2016). We experimentally determined MIC ranges of antibiotics for both *E. coli* MG1655 and *P. aeruginosa* (PA01) in the current study, using a method based on serial two-fold dilutions of antibiotics in 2 ml LB media in 14 ml test tubes (Andrews, 2001). The MIC ranges of *E. coli* MG1655 were found to be 3.125–6.25 μg/ml for ampicillin and 0.039–0.078 μg/ml for ofloxacin (Supplementary Table S1). The MIC range of *P. aeruginosa* (PA01) was found to be 0.3125–0.625 μg/ml for ofloxacin (Supplementary Table S1).

### Media, Chemicals, and Culture Conditions

All chemicals were purchased from Fisher Scientific (Atlanta, GA), VWR International (Pittsburg, PA) or Sigma Aldrich (St. Louis, MO). Luria-Bertani (LB) liquid media, prepared from its components (5 g yeast extract, 10 g tryptone and 10 g sodium chloride in 1 L ultra-pure DI water), and Mueller-Hinton (MH) liquid media (21 g premixed MH in 1 L ultra-pure DI water) were used to grow *E. coli* and *P. aeruginosa*, respectively. LB agar media (40 g premixed LB agar in 1 L ultra-pure DI water) and MH agar media (38 g premixed MH agar in 1 L ultra-pure DI water) were used to enumerate the colony forming units (CFUs) of *E. coli* and *P. aeruginosa* strains, respectively (Keren et al., 2004a; Amato et al., 2013; Orman and Brynildsen, 2015). Phosphate Buffered Saline (PBS) solution was used to wash the cells to remove the chemicals and antibiotics before plating them on agar media. For persister assays, 5 μg/ml ofloxacin and 200 μg/ml ampicillin were used (Keren et al., 2004a, b; De Groote et al., 2009; Allison et al., 2011). For selection and retention of plasmids in bacteria, 50 μg/ml kanamycin was added in culture media (Orman and Brynildsen, 2015). To induce fluorescent protein expression, 1 mM IPTG was used (Orman and Brynildsen, 2015).

Primary drug screening was performed using Phenotype MicroArrays (PM11-20) in 96-well plate formats, containing various chemicals including FDA approved compounds (Biolog Inc., Hayward, CA). Eleven chemicals, identified as initial hits, were purchased separately for further investigation: amitriptyline hydrochloride (Fisher catalog# 50-144-4347), trifluoperazine hydrochloride (Fisher catalog# T28495G), thioridazine hydrochloride (Fisher catalog# 30-705-0), CPZ (Fisher catalog# C24815G), CCCP (Fisher catalog# 04-525-00), protamine sulfate (Fisher catalog# AAJ6292609), promethazine hydrochloride (Fisher catalog# P2029100G), dodecyltrimethyl ammonium bromide (Fisher catalog# D146825G), triclosan (Fisher catalog# 64-795-01GM), polymyxin B Sulfate (Fisher catalog# 52-915-GM) and poly-L-lysine hydrochloride (VWR catalog# IC15269080). All chemicals were dissolved in ultra-pure DI water followed by filter-sterilization, except for CCCP and triclosan which were dissolved in DMSO. All LB and MH media were sterilized by autoclaving. Overnight pre-cultures were prepared in 14-ml falcon tubes containing 2 ml LB broth inoculated from a 25% glycerol (−80°C) cells stock and grown for 24 h at 37°C with shaking (250 rpm). Overnight pre-cultures were diluted in fresh 2 ml media in 14-ml test tubes or 25 ml media in 250-ml baffled flasks for the subsequent assays as described below. Cells cultured in the presence of the solvent (DI water or DMSO) served as controls when the cultures were treated with chemical inhibitors.

### Cell Growth and Persister Assays

Overnight pre-cultures were diluted 1000-fold in 2 ml fresh LB media in test tubes and grown at 37°C with shaking (250 rpm). Cell growth was monitored up to 24 hours by measuring optical density at 600 nm wavelength (OD_600_) with a plate reader (Varioskan LUX Multimode Microplate Reader, Thermo Fisher, Waltham, MA, United States) for selected time points. When required, cells were treated with chemicals before their transition to stationary phase (*t* = 5 h). At late-stationary phase (*t* = 24 h), cells were diluted in 2 ml fresh media (yielding ∼5 × 10^7^ cells/ml) with antibiotics (5 μg/ml ofloxacin or 200 μg/ml ampicillin) in test tubes and incubated at 37°C with shaking (250 rpm). At designated time points (*t* = 0, 1, 2, 3, 4, 5, and 6 h), 100 μl samples were collected and washed with PBS to dilute the antibiotics to sub-MIC levels, followed by resuspension in 100 μl of PBS. Ten microliters of the cell suspension were serially diluted and plated on LB agar media to enumerate CFUs. The remaining 90 μl cell suspensions were also plated to increase the limit of detection. The agar plates were incubated at 37°C for 16 h, which was found to be sufficient for *E. coli* colony formation (data not shown).

### Redox Sensor Green Dye Staining

Stationary-phase reductase and ETC activities were measured with Redox Sensor Green (RSG) dye (Thermo Fisher, catalog# B34954) according to manufacturer’s instructors. Cells at late-stationary phase (*t* = 24 h) were diluted 100-fold in 1 ml PBS in flow cytometry tubes (5 ml round bottom falcon tubes, size: 12 × 75 mm) and stained with RSG at 1 μM concentration. For negative controls, CCCP (10 μM) was added in the cell suspensions 5 min before RSG staining to disrupt membrane electron transport. Mid-exponential-phase cells were used as positive controls (Orman and Brynildsen, 2013a; Kim et al., 2018). Samples were incubated at 37°C for 10 min before analyzing with a flow cytometer (NovoCyte Flow Cytometer, NovoCyte 3000RYB, ACEA Biosciences Inc., San Diego, CA, United States). Forward and side scatter parameters of unstained controls were used to gate the cell populations on flow diagrams. Cells were excited at 488 nm with solid-state laser, and green fluorescence was collected with a 530/30 bandpass filter. To analyze the effect of chemical inhibitors (e.g., CPZ) on stationary-phase cell metabolism, cells at *t* = 5 h were treated with the chemicals at indicated concentrations, and RSG staining was performed at *t* = 24 h as described above.

### Monitoring Cell Division and Quantifying VBNC Cells

To monitor cell division and quantify non-growing cell subpopulations, inducible fluorescent protein (*mCherry*) expression systems were used. Overnight pre-cultures of *E. coli* MO strain were diluted 1000-fold in 2 ml LB media with 1 mM IPTG (to induce *mCherry*) in test tubes and grown as described above. It was previously verified that *mCherry* expression cassette or overexpressing *mCherry* did not affect the *E. coli* persistence (Orman and Brynildsen, 2013a, b, 2015). If necessary, cells at *t* = 5 h were treated with chemical inhibitors (e.g., CPZ) at indicated concentrations. At *t* = 24 h, *mCherry*-positive cells were collected, washed twice with PBS to remove the IPTG and chemicals, resuspended (100-fold) in 25 ml fresh LB media without inducer in 250 ml baffled flasks and cultured at 37°C with shaking (250 rpm). At designated time points (*t* = 0, 1, 2, and 2.5 h), cells were collected, washed and resuspended in PBS to measure their fluorescent protein content with a flow cytometer. When necessary, cells were further diluted in PBS to reach a desired cell density for the flow analysis (10^6^–10^7^ cells/ml). Cell division was monitored by measuring the dilution rate of fluorescent protein at single cell level. At *t* = 0 h, all cells exhibited a high level of red fluorescence, which declined as the cells divided, except in a small subpopulation whose fluorescence remained constant due to the lack of division (*t* = 2.5 h). Given that ampicillin only targets the proliferating cells, the cultures were further challenged with ampicillin (200 μg/ml) to quantify VBNC and persister cells in non-growing cell subpopulations. Using LIVE/DEAD staining, FACS and clonogenic survival assays, we previously showed that antibiotic sensitive cells were rapidly lysed by ampicillin while VBNC and persister cells remained intact (Orman and Brynildsen, 2013b). The intact cells were quantified using the volumetric-based cell counting feature of the NovoCyte Flow Cytometer. Persisters were quantified by enumerating the CFUs after plating the ampicillin treated cultures as described above. Intact cells that did not form colonies on standard medium were classified as VBNC cells (Roostalu et al., 2008; Jõers et al., 2010; Oliver, 2010; Luidalepp et al., 2011; Orman and Brynildsen, 2013b; Ayrapetyan et al., 2015). All samples were assayed with lasers emitting at 561 nm and red fluorescence was collected by 615/20 nm bandpass filter.

### Fluorescent Protein Degradation Assay

Overnight pre-cultures of *E. coli* MG1655 harboring pQE-80L-*gfp*-*ssrA* were inoculated (1:1000-fold) in 2 ml LB in test tubes, grown in the presence of IPTG (to induce SsrA-tagged GFP) until *t* = 5 h. Then, the cells were washed to remove the inducer, resuspended in filter-sterilized 2 ml spent medium (obtained from cultures grown under identical conditions without the inducer) and cultured in test tubes at 37°C with shaking (250 rpm). When necessary, cell suspensions were treated with chemical inhibitors. At designated time points, 200 μl samples were collected to measure their GFP levels with a plate reader. Excitation and emission wavelengths for GFP detection was 485 nm and 511 nm, respectively.

### Chemical Screening

**FIGURE 1:**
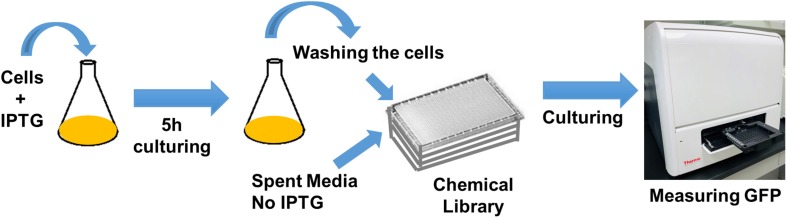
High-throughput drug screening approach to identify chemical compounds that inhibit GFP degradation. Cells expressing SsrA-tagged GFP at *t* = 5 h were re-suspended in spent medium, without inducer, transferred to 96-well PM plates containing the chemical library, covered with sterile, oxygen-permeable sealing membranes, and cultured in a shaker for 4 h. GFP measurements taken at 4 h were normalized to those taken at 0 h (after transferring the cells to plates).

Cells expressing SsrA-tagged GFP (grown in 25 ml LB with IPTG in 250 ml baffled flasks) at *t* = 5 h were washed, resuspended in spent medium (without inducer), transferred to 96-well PM plates (100 μl per well) containing the chemical library, covered with sterile, oxygen-permeable sealing membranes, and cultured in a shaker at 37°C and 250 rpm (Figure 1). GFP levels were monitored for 4 h using a plate reader, with cells cultured in the presence of the solvent serving as the negative controls, and those with CPZ as a positive control. GFP measurements taken at 4 h were normalized to those taken at 0 h to eliminate any variations in initial cell concentrations. *Z*-score method, calculated from the mean and the standard deviation of all measurements within the plate (Malo et al., 2006) was used to determine initial hits:

$$Z-\text{score}=\frac{X_{i}-\bar{X}}{S_{X}}$$

where *X*_*i*_ is the measurement (normalized) of the *i*th compound, $\bar{X}$and *S*_*X*_ are the mean and the standard deviation of all measurements. An absolute *Z*-score of ≥2, which correlates to a *P*-value of 0.045 (Martin et al., 2014), was assumed to be the threshold for hit detection. We note that each plate contains four different concentrations for each compound (information on these concentrations was not disclosed by the company). *Z*-scores were calculated for each concentration set. The initial hits were selected among the chemicals that successfully inhibited GFP degradation (*Z*-score ≥ 2) with at least two different concentrations.

Assay validation was evaluated by *Z*-factor calculated from the mean and standard deviation values of the positive (p) and the negative (n) control plates, as follows:

$$Z-\text{factor}=1-3\times \frac{\left(S_{p}+S_{n}\right)}{\left|{\bar{X}}_{p}-{\bar{X}}_{n}\right|}$$

A *Z*-factor between 0.5 and 1.0 indicates that the proposed assay is robust and reliable (Zhang et al., 1999).

### Validating the Selected Chemicals

To fully assess their utility and effectiveness, the selected chemical hits were analyzed at various concentrations with the aforementioned assays. Overnight pre-cultures of *E. coli* strains (WT, MO or cells expressing SsrA-tagged GFP) were inoculated (1:1000-fold) in 2 ml LB (IPTG was added for the cells harboring inducible fluorescent proteins) in test tubes and cultured as described. Cells at *t* = 5 h were treated with chemicals at indicated concentrations. Fluorescent protein degradation assays throughout the stationary phase after the treatments were performed for the cultures of *E. coli* cells expressing SsrA-tagged GFP; persister and cell survival assays at late-stationary phase (*t* = 24 h) were performed for WT cultures; and finally, cell division assays at late-stationary phase were performed for the *E. coli* MO cultures.

### *Pseudomonas aeruginosa* Persister Assay

Overnight pre-cultures of *P. aeruginosa* (PA01) were inoculated (1:1000-fold) in 2 ml MH broth in test tubes and cultured as described above. At *t* = 5 h, cells were treated with chemicals at indicated concentrations. At *t* = 24 h, cells were washed to remove chemicals and inoculated (1:100-fold) in 1 ml MH broth followed by ofloxacin (5 μg/ml) treatment. At *t* = 0 (before ofloxacin treatments), 10 microliter cell suspensions were serially diluted and spotted on MH agar media to enumerate initial CFUs, which enables us to assess the impacts of chemical treatments on *P. aeruginosa* (PA01) cell viability. To enumerate persister levels at *t* = 6 h, ofloxacin treated cultures were washed, serially diluted and plated on MH agar media. The plates were incubated for 20 h at 37°C. Twenty-hour incubation was found to be sufficient for *P. aeruginosa* (PA01) colony formation (data not shown).

### Statistical Analysis

To compare the biphasic kill curves and evaluate the statistical significance, we used a non-linear model described by Windels et al., 2019:

$${\text{Log}}_{10}\,{\text{N}}_{\text{i }}={\text{Log}}_{10}\,\left\{\left({\text{N}}_{0}-{\text{P}}_{0}\right)\,{\text{e}}^{-{\text{k}}_{\text{n}}\,\text{t}}+P_{0}\,{\text{e}}^{-{\text{k}}_{\text{p}}\,\text{t}}\right\}$$

where, N_0_ = initial number of normal cells (CFU/ml), P_0_ = initial number of persister cells (CFU/ml), k_*n*_ = normal cell killing rate, k_*p*_ = persister cells killing rate, *t* = time (h), and *N*_*i*_ = number of survived cells (CFU/ml) at t. GraphPad Prism 8.3.0 (GraphPad Prism version 8.3.0 for Windows, GraphPad Software, La Jolla, California, United States)^1^ was used for the non-linear regression analysis, where each replicate is considered as a random component, and each treatment is considered as a fixed component. *F*-statistics was used to test the null (one curve for all experimental groups) and the alternative hypothesis (different curve for each experimental group). Quantile-quantile (QQ) normality plots indicated that persister data sets (control and treatment groups) can be assumed to be normally distributed (Supplementary Figure S11). These figures were generated for each experimental group by plotting actual residuals vs. predicted residuals sampled from a Gaussian distribution with a built-in function in GraphPad Prism.

To perform pairwise or group comparisons, we have used one-way ANOVA with Dunnett’s posttest. At least three independent biological replicates were performed for all experiments. Each data point in figures is represented by mean ± standard deviation. *P*-value threshold was chosen as ^∗^*P* < 0.05 or ^∗∗^*P* < 0.0001.

## RESULTS

### Chlorpromazine Hydrochloride (CPZ) Pretreatment Can Reduce *E. coli* Persistence

**FIGURE 2:**
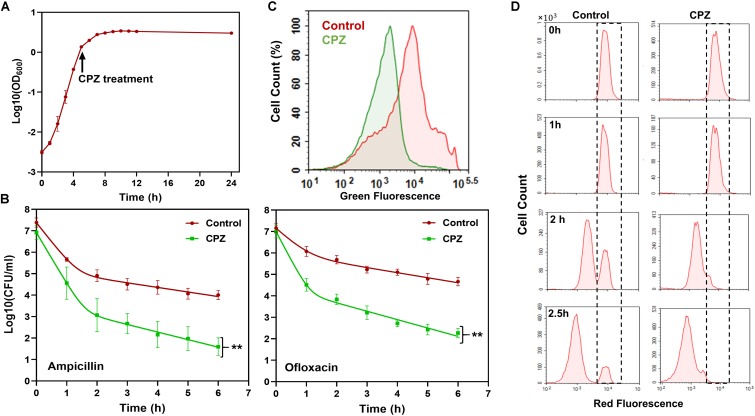
CPZ treatment reduced stationary-phase persistence, redox activities and non-growing cell formation. **(A)** CPZ treatment. Cells were treated with 0.25-mM CPZ at *t* = 5 h or left untreated (control); cells in late-stationary phase (*t* = 24 h) were then washed to remove the inhibitors to measure persister and non-growing cell levels as well as cellular redox activities. Cell growth was monitored with OD_600_ measurements. **(B)** Persister levels of CPZ-treated cultures. Untreated or CPZ-treated cells in late-stationary phase (*t* = 24 h) were washed to remove the chemicals and resuspended (1:100-fold) in fresh media with antibiotics for persister quantitation (Number of biological replicates, *N* = 6). The solid lines represent biphasic kill curve fits. **(C)** RSG staining of CPZ-treated or untreated late-stationary-phase cells. Untreated or CPZ-treated cells in late-stationary phase (*t* = 24 h) were washed to remove the chemicals, and resuspended in PBS with RSG (*N* = 6). All 6 biological replicates consistently resulted in similar trends. **(D)** Flow-cytometry histograms for the non-growing cell quantification in CPZ-treated cultures. Cells (harboring an IPTG inducible mCherry expression cassette) were treated with 0.25-mM CPZ at *t* = 5 h or left untreated (control) in the presence of IPTG; cells in late-stationary phase (*t* = 24 h) were washed to remove the chemicals and diluted in fresh media without IPTG. Division at the single-cell level was monitored by flow cytometry during exponential-growth phase. A representative biological replicate is shown here. All 3 biological replicates consistently resulted in similar trends. **Statistical significance between control (untreated) and CPZ treatment group (*P*-value<0.0001, *F*-Statistics).

To test if targeting one of the key components, i.e., ATP synthase, in cell metabolism can reduce persister formation in stationary phase, we first treated *E. coli* cultures with CPZ throughout stationary phase (Figure 2A), and then diluted cells into fresh media with antibiotics (ampicillin and ofloxacin) for persister cell quantification. As expected, we were able to reduce *E. coli* persistence in fresh media (Figure 2B) when the cells were pretreated with CPZ at a concentration that did not affect stationary-phase-cell survival (Supplementary Figure S1A). We note that cells were not treated with antibiotics directly in stationary-phase cultures, as normal cells are intrinsically tolerant in these cultures to ampicillin whose mechanism of action requires cell growth. The stationary-phase cells were first washed to remove the metabolic inhibitors, transferred to fresh medium, and then treated with antibiotics to stimulate non-persister cell killing.

Pretreatment with CPZ also reduced stationary-phase-metabolic activities, measured by RSG dye (Figure 2C and Supplementary Figure S1B). RSG can readily penetrate bacteria and yield green fluorescence when reduced by bacterial reductases; hence, fluorescent signals produced by RSG correlate with cellular metabolic activities (Supplementary Figure S2). Overall, these results verify that bacterial metabolism is a rich source of novel strategy to eliminate persisters.

### CPZ Pretreatment Reduced Non-growing Cell Levels in Exponential Phase Cultures

Although some persistent infections are associated with clinically apparent chronic symptoms, some cases are asymptomatic for a long period of time (e.g., a decade) and can develop clinically significant diseases at later times (Grant and Hung, 2013). The bacteria causing asymptomatic infections can be present within the host system in a non-replicating or slowly replicating state (generally referred to as “viable but non-culturable” or VBNC state) and cannot be easily cultured *in vitro* (Oliver, 2010; Ayrapetyan et al., 2015). We and others have previously shown that antibiotic-treated cultures have many more VBNC cells than persisters (∼2-log-fold more) (Roostalu et al., 2008; Jõers et al., 2010; Luidalepp et al., 2011; Orman and Brynildsen, 2013b). Both persister and VBNC cells are stained as live, retain metabolic activity, and often appear as non-growing during the antibiotic treatment (Orman and Brynildsen, 2013b). The only means to distinguish these subpopulations lies in the ability of persisters but not VBNC cells to recolonize in standard culture media in the absence of antibiotics. To determine whether the CPZ pretreatment eliminate VBNC, we used our published method where we monitor cell proliferation via an inducible fluorescent protein (*mCherry*) expression cassette (Orman and Brynildsen, 2013a, b, 2015), in which *mCherry*-positive cells from late-stationary-phase cultures are inoculated in fresh medium in the absence of inducer (Figure 2D, *t* = 0). Flow cytometry reveals ongoing cell division as a dilution of *mCherry*, whereas the fluorescence levels are maintained in the non-proliferating subpopulation (Figure 2D, WT at *t* = 2.5 h, highlighted with dashed lines). Although persisters were shown to be enriched in this subpopulation (Orman and Brynildsen, 2013a), most of these non-growing cells were identified as VBNC cells (Orman and Brynildsen, 2013a) (Supplementary Figures S3A,B), which were not detected in the CPZ treated cultures (Figure 2D, CPZ at *t* = 2.5 h, and Supplementary Figure S3A). The reduction in both persister and VBNC cell levels in the CPZ treated cultures points out these two phenotypes may be related. Consistent with the general notion in the field, it is possible that persistence may be a transitory phase leading to the VBNC state (Ayrapetyan et al., 2015). Whether persistence contributes to the accumulation of VBNC cells due to the catabolism of intracellular components warrants further investigation.

### High-Throughput Screening Detected Chemical Compounds That Target *E. coli* Metabolism and Persistence

**FIGURE 3:**
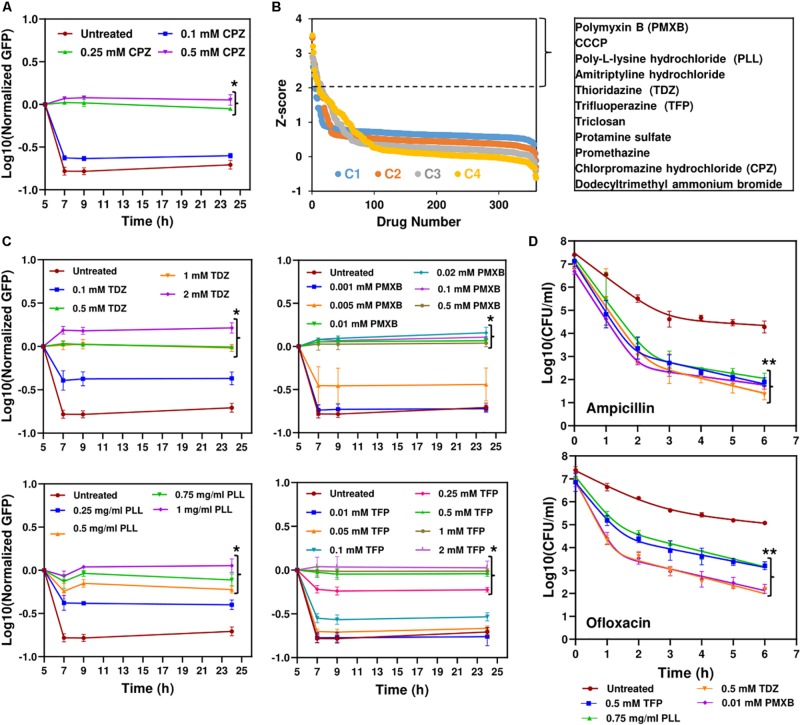
High-throughput drug screening detected chemical compounds that inhibit persistence. **(A)** Inhibition of GFP degradation with CPZ treatment at indicated concentrations. Cells expressing SsrA-tagged GFP were grown to until *t* = 5 h in the presence of IPTG (inducer) and then re-suspended in a filter-sterilized spent medium (without inducer and obtained from the cultures grown under identical conditions) and immediately treated with CPZ to inhibit cell metabolism and protein degradation. Green fluorescence levels were measured and normalized to their initial levels (*t* = 5 h, before CPZ treatment) to determine GFP degradation. Background fluorescence was determined using cells with empty vectors (*N* = 3). **(B)** The *Z*-scores calculated for the chemical compounds at four different concentrations (C_4_ > C_3_ > C_2_ > C_1_). Note that these concentrations were not disclosed by Biolog, Inc. The initial hits tabulated were selected among the chemicals that have *Z*-scores ≥2 with at least two different concentrations. **(C)** Inhibition of GFP degradation by the identified drugs. The selected hits were analyzed in depth at various concentrations to select the drugs that can reduce GFP degradation without affecting the *E. coli* cell viability (Supplementary Figure S6). Cells were treated with these drugs at *t* = 5 h, at indicated concentrations, and then, GFP measurements were performed at indicated time points. **(D)** Persister levels of the cell cultures pretreated with the chemical hits. Cells at *t* = 5 h were treated with selected drugs at indicated concentrations. At late-stationary phase (*t* = 24 h), treated cells were washed to remove the drugs and diluted (1:100-fold) in fresh media with indicated antibiotics for persister enumeration (*N* = 3). The solid lines represent biphasic kill curve fits. *Statistical significance between drug-treated and untreated cultures for the last time points (*P* < 0.05, one-way ANOVA with Dunnett’s posttest). **Statistical significance between control (untreated) and treatment groups (*P*-value<0.0001, *F*-Statistics). CPZ, Chlorpromazine hydrochloride; PMXB, Polymyxin B; PLL, Poly-L-lysine; TDZ, Thioridazine; TFP, Trifluoperazine.

To directly measure protein degradation rates in stationary-phase cultures, we previously developed an assay (Orman and Brynildsen, 2015) using GFP that is linked to a short peptide degradation tag (11 amino acid residues), SsrA, to mark it for degradation by cellular proteases, mainly ClpAP and ClpXP (Lon, Tsp and FtsH are also known to target the ssrA sequence) (Herman et al., 1998; Flynn et al., 2001; Spiers et al., 2002; Choy et al., 2007). Although we note that self-digestion is a complex network orchestrated by many degradative enzymes (proteases, RNases and toxins), we showed that metabolic inhibitors can prevent digestion of endogenous proteins by reducing cell metabolism (Orman and Brynildsen, 2015, 2016). As expected, CPZ treatment suppressed degradation of this tag in stationary-phase cultures (Figure 3A and Supplementary Figure S4A), potentially by reducing stationary-phase-metabolic activities (Figure 2C and Supplementary Figure S1B). To test whether this straightforward system can identify additional therapeutics to eliminate persisters, we used a small library (Biolog Phenotype Arrays), containing antibiotics and other FDA approved drugs among ∼360 known chemical compounds in 96-well plate formats. Cells expressing SsrA-tagged GFP were transferred to the phenotype arrays without inducer before entering stationary phase, and cultured under the conditions studied here (see section “Materials and Methods”). GFP levels were monitored using a plate reader, with cells cultured in the presence of the solvent serving as the negative controls, and those with CPZ as a positive control. Our data verify that GFP in negative controls is degraded within 4 h (Supplementary Figure S4A). The *Z*-factor (predicted by analysis of test plates with negative and positive controls, as described in section “Materials and Methods”) was calculated to be 0.836, which indicates the robustness of our methodology (Malo et al., 2006). Given that each plate contains four different concentrations for each compound (information on these concentrations was not disclosed by the company), the initial hits were selected among the chemicals that successfully inhibited GFP degradation (*Z*-score ≥ 2) with at least two different concentrations (Figure 3B and Supplementary Figure S4B). As expected, CPZ, which is one of the 360 chemical compounds tested, was identified as a positive hit, verifying that our method can detect potential metabolic inhibitors (Figure 3B and Supplementary Figure S4B). To determine chemical inhibitors that specifically target persister metabolism, the identified hits were further analyzed in additional rounds of screening to determine concentrations that lead to complete inhibition of GFP degradation without affecting the stationary-phase-cell viability (Figure 3C and Supplementary Figures S5, S6). We identified that CCCP, polymyxin B, poly-L-lysine, thioridazine (TDZ), and trifluoperazine (TFP) did not drastically affect the cell viability at the inhibitory concentrations for GFP degradation (Supplementary Figure S6), and four drugs, except CCCP, were able to reduce persistence (Figure 3D and Supplementary Figure S7). We note that FCCP was also identified as an initial hit but we did not investigate this compound further as it is structurally very similar to CCCP. Both FCCP and CCCP are extensively used as an ionophore (Terada, 1990; Woronowicz et al., 2015).

**FIGURE 4:**
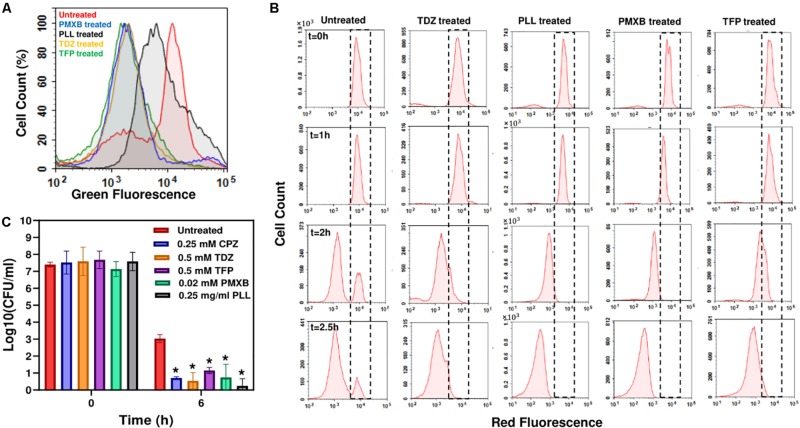
Drug treatments reduced stationary-phase-redox activities and non-growing cell formation in *E. coli*, and persister levels in *P. aeruginosa*. **(A)** RSG staining of drug-treated or untreated late-stationary-phase *E. coli* cells. Cells were treated with the drugs at *t* = 5 h, and RSG staining was performed at late-stationary phase (*t* = 24 h). Drug concentrations: 0.5 mM Thioridazine (TDZ); 0.75 mg/ml Poly-L-lysine (PLL); 0.01 mM Polymyxin B (PMXB); 0.5 mM Trifluoperazine (TFP) (*N* = 6). **(B)** Flow-cytometry histograms for the non-growing cell quantification in drug-treated *E. coli* cultures. A representative biological replicate is shown here. All three biological replicates consistently resulted in similar trends. Drug concentrations are the same as those provided in panel **(A)**. **(C)** Persister levels in *P. aeruginosa* cultures treated with the chemical hits. Cells at *t* = 5 h were treated with the selected drugs or left untreated (control); cells in late-stationary phase were then washed to remove the inhibitors and re-suspended in fresh media with ofloxacin (effective for *P. aeruginosa*) for persister assays. Cells were plated for CFU enumeration before and after the ofloxacin treatments to assess the effects of drugs on *P. aeruginosa* cell viability and persistence, respectively (*N* = 6). *Statistical significance between drug-treated and untreated cultures (*P* < 0.05, one-way ANOVA with Dunnett’s posttest).

Both TDZ and TFP fall under the category of phenothiazine antipsychotic drugs, which are tricyclic compounds structurally similar to chlorpromazine. These drugs have been shown to reduce or inhibit NADH2-menaquinone-oxidoreductase and succinate dehydrogenase activities as well as NADH/NAD+ ratios (Boshoff et al., 2004; Weinstein et al., 2005; Yano et al., 2006), consistent with our RSG staining results provided in Figure 4A and Supplementary Figure S8. We observe similar reduction in stationary-phase cellular redox activities after polymyxin B and poly-L-lysine treatments (Figure 4A and Supplementary Figure S8). These cationic peptides were shown to electrostatically bind to bacterial cells that leads to possible disruption of the bacterial membranes and membrane potential (Domingues et al., 2012; Li et al., 2014), which explains the observed reduction in bacterial redox activities (Figure 4A and Supplementary Figure S8). Treating the stationary-phase cells with these four chemicals further reduces VBNC formation (Figure 4B and Supplementary Figure S9), consistent with the results obtained from CPZ treatments. Overall, these results strongly support that the identified drugs eliminate bacterial persistence by inhibiting stationary-phase metabolism.

### The Identified Drugs Can Reduce *Pseudomonas aeruginosa* Persistence

Our previous results indicate that persistence is facilitated by a self-digestion mediated metabolic futile cycle, wherein energy derived from catabolism is dissipated through continuous degradation of cellular components (Orman and Brynildsen, 2015, 2016). This process also introduces a self-inflicted damage in the cells that transiently repressed the cellular functions targeted by antibiotics (Orman and Brynildsen, 2015). The identification and characterization of the main components of this metabolic cycle may provide a global treatment approach as it can be an evolutionarily conserved process that may occur in many prokaryotes and eukaryotes and enable survival under stressful conditions (such as nutrient depletion, aging and overpopulation) via the recycling of essential energy molecules. When we similarly tested the identified chemicals on *P. aeruginosa* (PAO1), we were able to substantially reduce *P. aeruginosa* persistence, suggesting the existence of similar mechanisms in other bacteria (Figure 4C and Supplementary Figure S10). These results provide further clinical relevance for the identified drugs, since *P. aeruginosa* is involved in many hospital-related biofilm infections and the predominant cause of morbidity and mortality in cystic fibrosis patients with compromised immune systems (Lyczak et al., 2000; Stover et al., 2000; Mulcahy et al., 2010).

## Discussion

As antibiotics are most effective against growing bacteria, the resistance of persisters has been attributed to transient growth inhibition. Experimental evidence supporting this hypothesis was obtained in 2004 by Balaban and colleagues, who showed bacteria that failed to replicate prior to an ampicillin challenge also failed to lyse or grow during antibiotic treatment, but began replicating once the antibiotic was removed (Balaban et al., 2004). This seminal study led to the model that persistence is a dormant phenotype, characterized by a depressed metabolism. However, recent evidence suggests persisters can harbor ETC activities associated with bacterial cytochromes and oxidoreductases (Orman and Brynildsen, 2015). They can consume certain carbon sources to generate proton motive force (PMF) (Allison et al., 2011; Orman and Brynildsen, 2013b), maintain high ATP levels (Baidoo et al., 2013; Radzikowski et al., 2016), and drive the futile production and degradation of RNA, leading to energy generation and dissipation (Mok et al., 2015). Interestingly, most persister-related genes identified so far either directly or indirectly modulate cell metabolism (Amato et al., 2014; Prax and Bertram, 2014; Radzikowski et al., 2017).

While inhibition of mRNA, protein, and ATP synthesis in exponentially growing cells was shown to induce cell dormancy and persistence (Correia et al., 2006; Kwan et al., 2013; Conlon et al., 2016; Shan et al., 2017), this may not be the case for stationary-phase cells. Our previous (Orman and Brynildsen, 2015) and the current study indicates that inhibiting stationary-phase metabolism can block the metabolic stresses and yield cells that are more capable of translation and replication and thus susceptible to cell death upon dilution in fresh medium. The role of metabolism is significant for bacteria, because bacteria must produce large amounts of energy and biosynthetic precursors to meet the metabolic demands of their rapid growth. The increased metabolism results in a number of metabolic stresses, including nutrient starvation, hypoxia, and oxidative stress (Matin, 1991; Nyström, 2004). These stresses promote intracellular degradation/damage maintained by degradative enzymes in stationary phase (Nyström, 2004) that may transiently repress the cellular functions targeted by antibiotics.

Our study provides strong support for the notion that stationary-phase metabolism is a rich source of novel strategies to eliminate the antibiotic-tolerant cells. The identified drugs (i.e., CPZ, TDZ, TFP, polymyxin B, and poly-L-lysine) are already known to target bacterial redox activities (Ohlow and Moosmann, 2011). CPZ, TDZ, and TFP are commonly known as first generation antipsychotic/neuroleptic drugs (Fenton et al., 2007; Ohlow and Moosmann, 2011; Dudley et al., 2017). Since they are the derivative of a heterocyclic phenothiazine, their mechanism of action is similar (Ohlow and Moosmann, 2011). The effectiveness of these drugs depends upon the ability to block dopamine receptors as the excessive dopamine is the main culprit of schizophrenia and other psychotic diseases (Girault and Greengard, 2004). These drugs were also shown to have antimicrobial activities. In *Mycobacterium tuberculosis*, phenothiazines inhibit cellular respiration, leading to depletion of ATP as well as the reduction of NADH/NAD+ and menaquinol/menaquinone ratios (Boshoff et al., 2004; Weinstein et al., 2005; Yano et al., 2006). Because of their ability to inhibit bacterial efflux pumps, they were also shown to enhance the sensitivity of *Staphylococcus aureus* to beta-lactam antibiotics (Amaral and Viveiros, 2012; Thorsing et al., 2013). Studies have shown that poly-L-lysine, which is a cationic polymer, can result in change of morphology in bacteria (Li et al., 2014). In addition, treatment with poly-L-lysine raises the electric conductivity of the bacterial cells which leads to possible disruption of the cytoplasmic membrane (Ye et al., 2013). Similarly, polymyxins consist of a polypeptide cationic ring made up of 8–10 amino acids, which have a disruptive physiochemical effect resulting in alternation of membrane permeability in bacteria (Evans et al., 1999). In addition, type II NADH-quinone oxidoreductases, which are integral part of ETC, has also been shown to be a secondary target sites of cationic peptides (Deris et al., 2014). Polymyxins have been administered for urinary tract infection, pneumonia, bacteremia, postoperative wound infections, abscesses, osteomyelitis (when given as an irrigation), and endocarditis (Evans et al., 1999).

Overall, we presented here a methodology that has been designed to challenge paradigms regarding metabolic dormancy in persisters, shed light on the often-overlooked metabolic processes of persister cells, develop a screening approach to identify metabolic inhibitors among a small library with FDA approved compounds, and integrate all proposed work to accelerate development of adjuvant therapies. Given that the cytotoxicity, cell permeability, solubility, and safety properties of FDA compounds have been well studied and documented during their preclinical and clinical research phases, discovering drugs that eliminate persisters among such libraries will have an enormous impact, because it will identify potential therapeutics that do not require the long laborious FDA approval process.

## Data Availability Statement

The raw data supporting the conclusions of this article will be made available by the authors, without undue reservation, to any qualified researcher.

## Author Contributions

SM, TH, AS, PK, and MO conceived and designed the study, analyzed the data and wrote the manuscript. SM, TH, AS, and PK performed the experiments.

## Conflict of Interest

The authors declare that the research was conducted in the absence of any commercial or financial relationships that could be construed as a potential conflict of interest.

## Footnotes

1 www.graphpad.com
