# Supplementary material for: Identifying Metabolic Inhibitors to Reduce Bacterial Persistence
Source: Front Microbiol. 2020 Mar 27;11:472. doi: 10.3389/fmicb.2020.00472 (PMC7118205; doi:10.3389/fmicb.2020.00472)
Supplement: Supplementary file 1 [file Presentation_1.pdf]

Supplementary Material

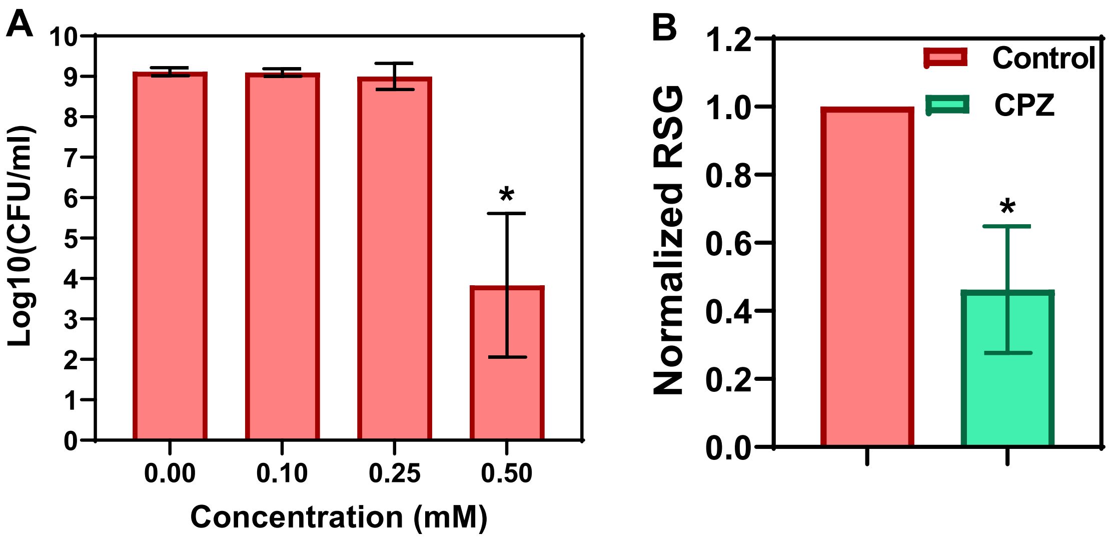

Fig. S1. Effects of chlorpromazine hydrochloride (CPZ) treatments on stationary-phase cell viability and redox activities. (A) Cells at t=5 h were treated with CPZ at indicated concentrations, and then, at late-stationary phase (t=24 h), washed to remove the chemicals and plated on agar media to assess the effects of CPZ treatments on cell viability. (B) Cells in late-stationary phase (t=24 h) were washed to remove the chemicals, and resuspended in PBS to stain with RSG. Fluorescence measurements were performed with a flow cytometry. Mean fluorescence values of cell populations were normalized to those obtained from untreated groups (control) (N=6).
* Statistical significance between drug-treated and untreated cultures ($P<0.05$, one-way ANOVA with Dunnett's posttest).

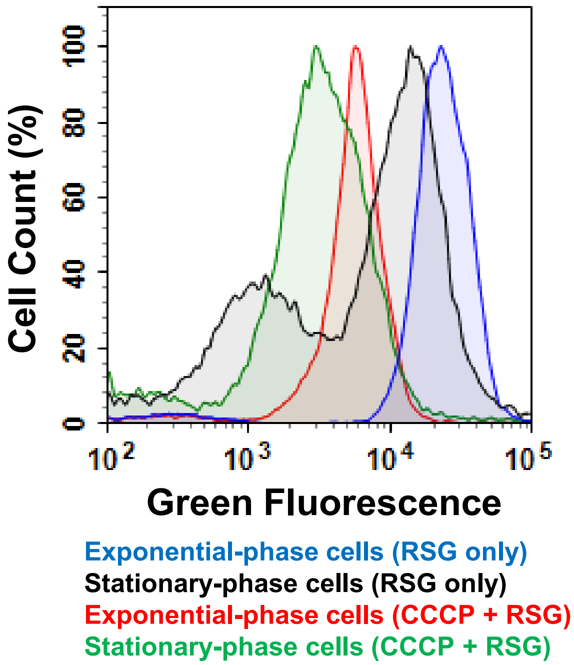

Fig. S2. RSG staining to measure bacterial reductase activity. Cells at mid-exponential phase (t=3 h) and late-stationary phase (t=24 h) were transferred to PBS and stained with RSG. For controls, cells were treated with a metabolic inhibitor, CCCP, as described in the manufacturer's protocol. Our data clearly shows that exponentially growing cells are highly metabolically active; this might explain why CCCP-treated exponential-phase cells have higher green fluorescence compared to that of stationary-phase cells.

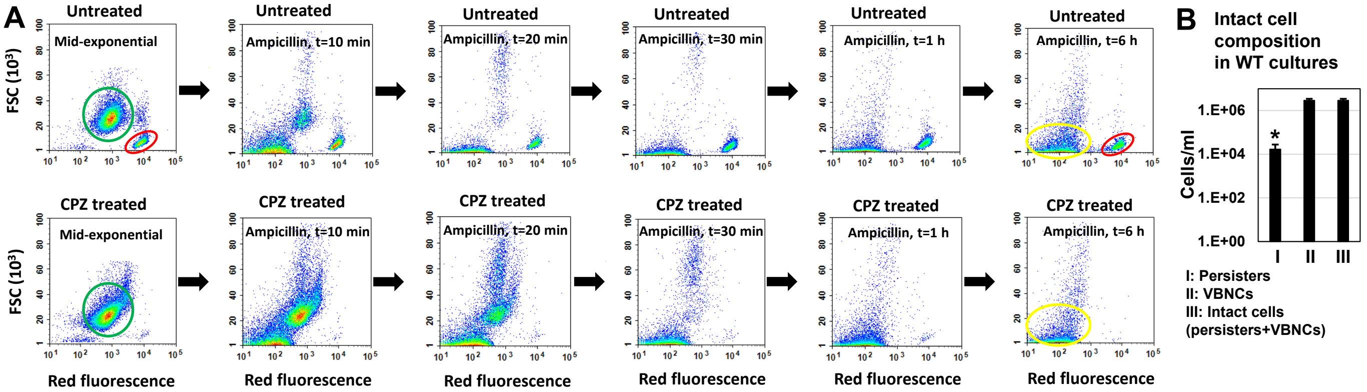

Fig. S3. Effects of CPZ treatments on VBNC cell levels. Overnight pre-cultures of MO cells (harboring an inducible mCherry expression system) were diluted 1000-fold in 2 ml LB in test tubes in the presence of inducer (1 mM IPTG) and cultured. Cells at t=5 h were treated with CPZ (0.25 mM) or left untreated. At t=24 h, cells were washed to remove the inducer and transferred to fresh media without IPTG to monitor cell growth. All cells exhibited high red fluorescence at t=0 h (Fig. 2D in the main text), and the red fluorescence signal declined as the cells divided (growing cells highlighted with a green circle), except for small subpopulations in which the fluorescence signal remained constant due to the lack of cell division (non-growing cells highlighted with a red circle). We note that the 1st column in Panel A corresponds to the last row of Fig. 2D in the main text. VBNC cell levels were determined with the ampicillin-treatment approach as described in Materials and Methods. When the cells at mid-exponential phase were treated with ampicillin, the growing cell subpopulations were lysed (debris highlighted with a yellow circle), however non-growing cells remained intact. Only a small fraction of intact cells (i.e., persister cells) colonized (panel B). The majority of intact cells were detected as VBNC cells (panel B). Non-growing cells were not detectable in CPZ treated cultures (panel A). Note that a representative biological replicate is shown here. All 3 biological replicates consistently resulted in similar trends.

* Statistical significance between intact cells, VBNCs and persisters ($P<0.05$, one-way ANOVA with Dunnett's posttest).

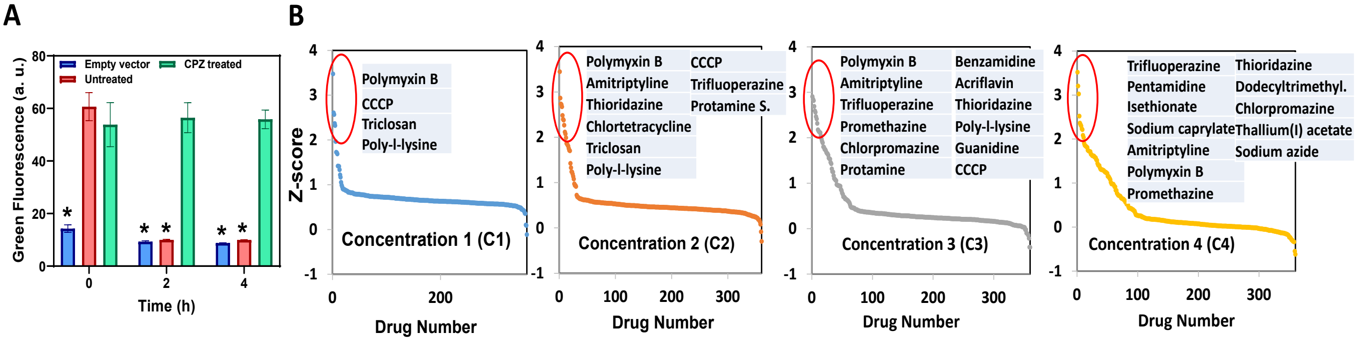

Fig. S4. Developing a chemical screening approach. (A) Stationary-phase GFP degradation was inhibited with CPZ treatment (0.25 mM). Cells harboring pQE-80L-*gfp-ssrA* were grown to t=5 h in the presence of IPTG (inducer) and then re-suspended in a filter-sterilized spent medium (obtained from the cultures grown under identical conditions without IPTG) and immediately treated with CPZ to inhibit cell metabolism and protein degradation. In the absence of inducer, GFP degradation was clearly observed in untreated cell cultures (no-CPZ treatment) whereas this degradation was not observed in CPZ-treated cultures within 4 hours. Background fluorescence was determined using cells with empty vectors (E.V.) (B) Cells at t=5 h, expressing SsrA-tagged GFP, were re-suspended in spent medium, without inducer, transferred to 96-well PM plates containing the chemical library, and cultured in a shaker for 4h. GFP measurements taken at 4 h were normalized to those taken at 0 h. The Z-scores were calculated for the chemical compounds at four different concentrations ($C_4 > C_3 > C_2 > C_1$). Chemicals with Z-scores >2 were tabulated for each concentration set. Eleven hits were selected among the chemicals that successfully inhibited GFP degradation (Z-score≥2) with at least two different concentrations (Fig. 3B in the main text).

* Statistical significance between drug-treated and untreated cultures (P<0.05, one-way ANOVA with Dunnett's posttest).

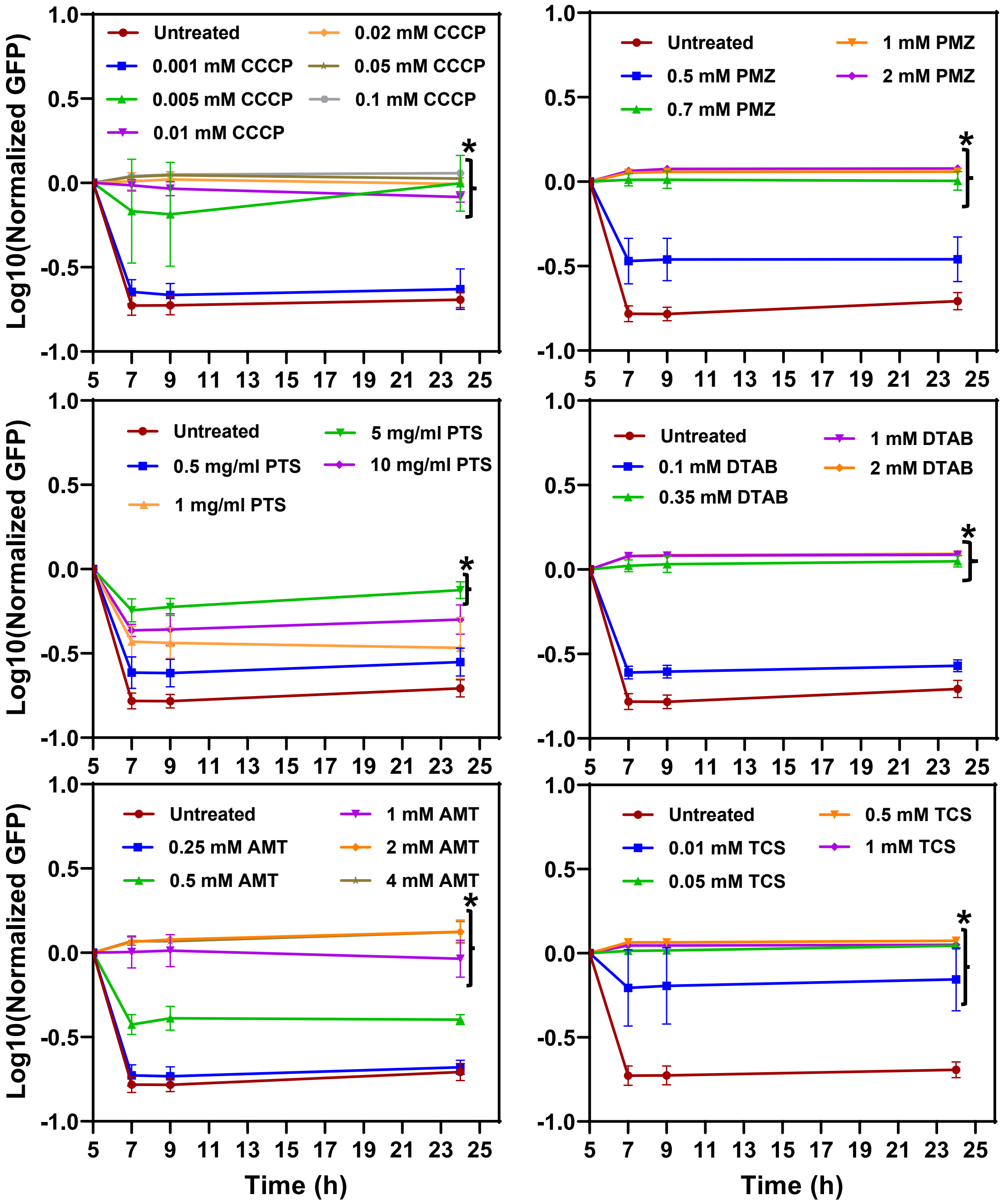

Fig. S5. Effects of drug treatments on degradable GFP levels. Cells were treated with hit drugs at t=5 h at indicated concentrations, and then, GFP measurements were performed at indicated time points. Eleven hits, selected based on the Z-scores analysis (Fig. 3B and Fig. S4B), were analyzed. Six chemicals are highlighted in this figure; the rest of the chemicals are highlighted in Fig. 3C in the main text. CCCP: Carbonyl cyanide m-chlorophenyl hydrazone; PMZ: Promethazine; PTS: Protamine Sulfate; AMT: Amitriptyline; DTAB: Dodecyltrimethylammonium bromide; TCS: Triclosan (N=3). * indicates the statistical significance between drug-treated and untreated cultures for the last time points ($P<0.05$, one-way ANOVA with Dunnett's posttest).

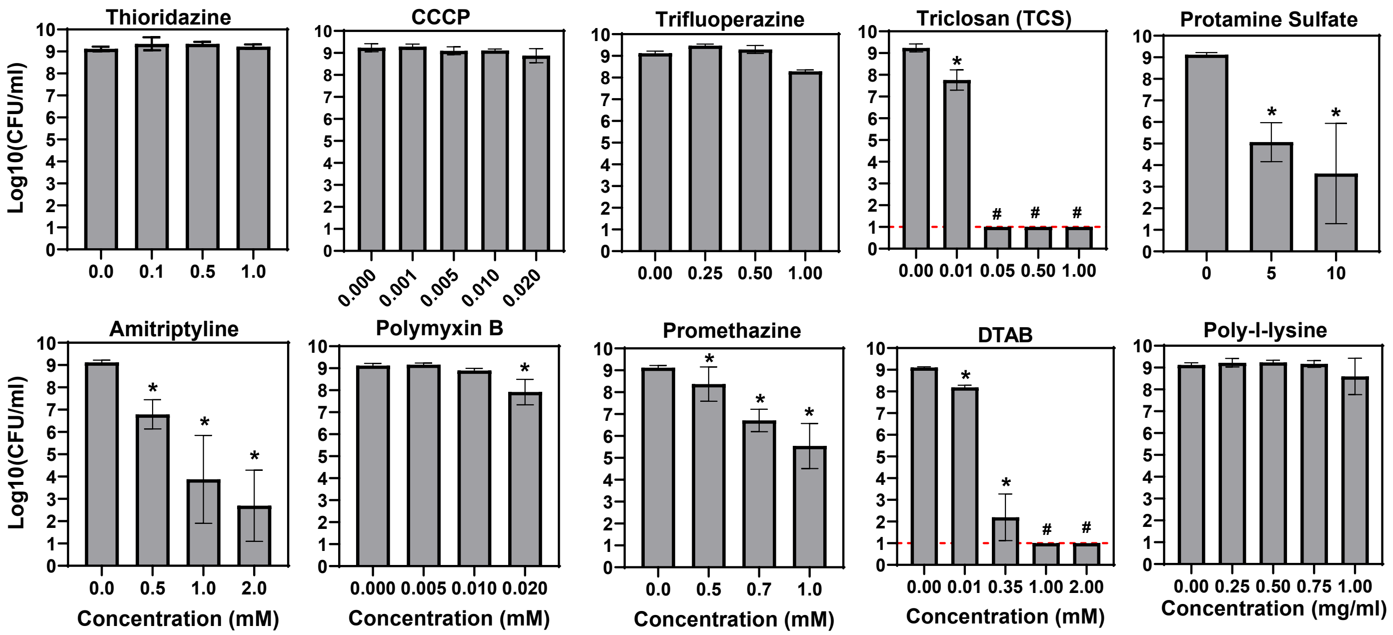

Fig. S6. Effects of chemical hits on cell viability. Cells at t=5 h were treated with chemical hits at various concentrations, and then, cells at late-stationary phase were washed to remove the chemicals and plated on agar media for CFU measurements. Among the chemicals tested, CCCP, Polymyxin B, Poly-L-lysine, Thioridazine, and Trifluoperazine did not affect the cell viability within a wide range of concentrations tested. Amitriptyline, Promethazine, Protamine Sulfate, Triclosan, and Dodecyltrimethyl ammonium bromide (DTAB) significantly reduced the stationary phase cell survival at concentration ranges that inhibit GFP degradation (N=3).
* Statistical significance between drug-treated and untreated cultures ($P<0.05$, one-way ANOVA with Dunnett's posttest).
# indicates the limit of detection.

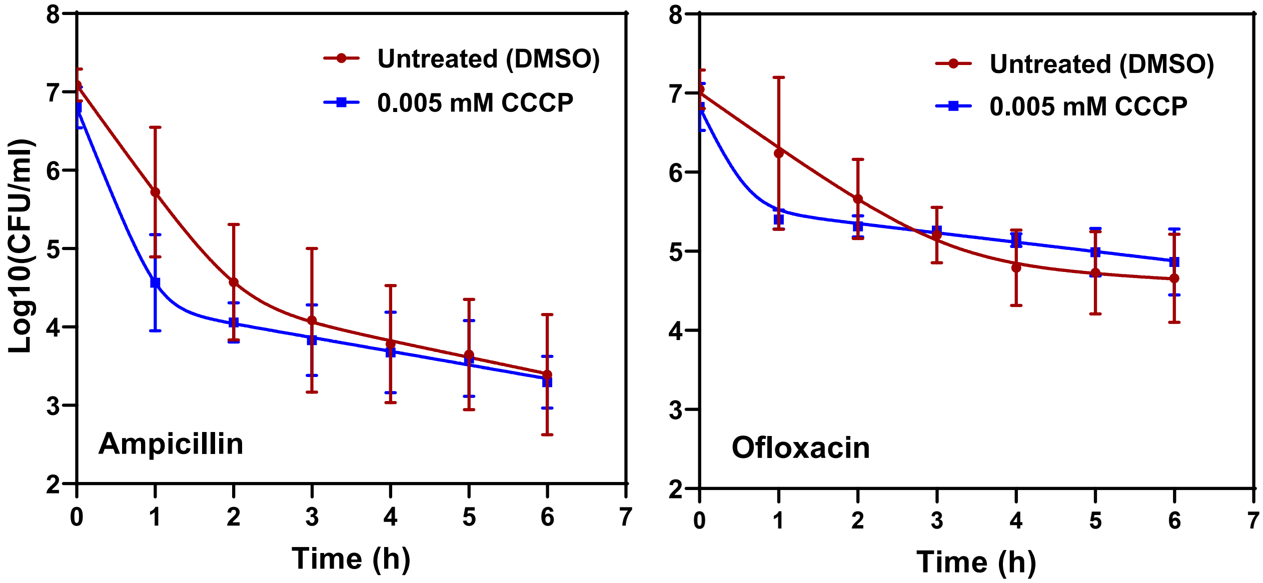

Fig. S7. Effects of CCCP treatments on persister levels. Cells at t=5 h were treated with the five chemicals (CCCP, Polymyxin B, Poly-L-lysine, Thioridazine, and Trifluoperazine, highlighted in Fig. S6) at concentrations that inhibit GFP degradation without affecting the stationary-phase-cell survival. Then, cells at late stationary phase were washed to remove the chemicals, transferred to fresh media and treated with ofloxacin and ampicillin. CCCP data is provided here; the data for the rest of the chemicals is provided in the main text (Fig. 3D). No statistical difference was detected between the control and CCCP treated cultures (N=3).

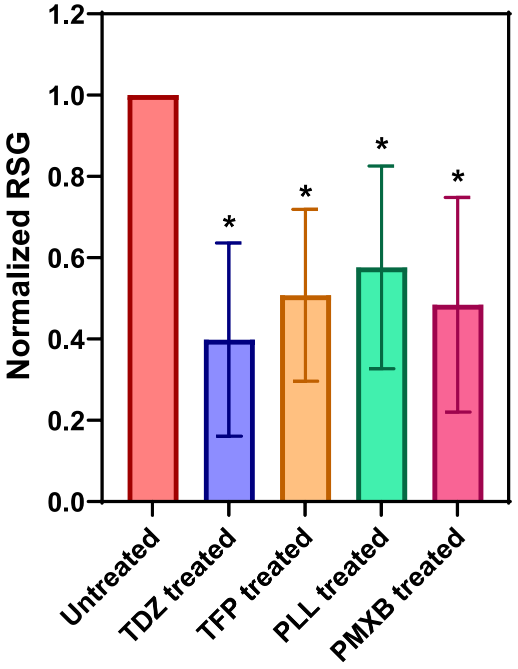

Fig. S8. RSG staining of drug-treated or untreated late-stationary-phase *E. coli* cells. Cells were treated with the drugs at t=5 h, and RSG staining was performed at late-stationary phase (t=24 h). Fluorescence measurements were performed with a flow cytometry. Mean fluorescence values of cell populations were normalized to those obtained from untreated groups (control). Drug concentrations: 0.5 mM Thioridazine (TDZ); 0.75 mg/ml Poly-L-lysine (PLL); 0.01 mM Polymyxin B (PMXB); 0.5 mM Trifluoperazine (TFP) (N=6).

* Statistical significance between drug-treated and untreated cultures ($P<0.05$, one-way ANOVA with Dunnett's posttest).

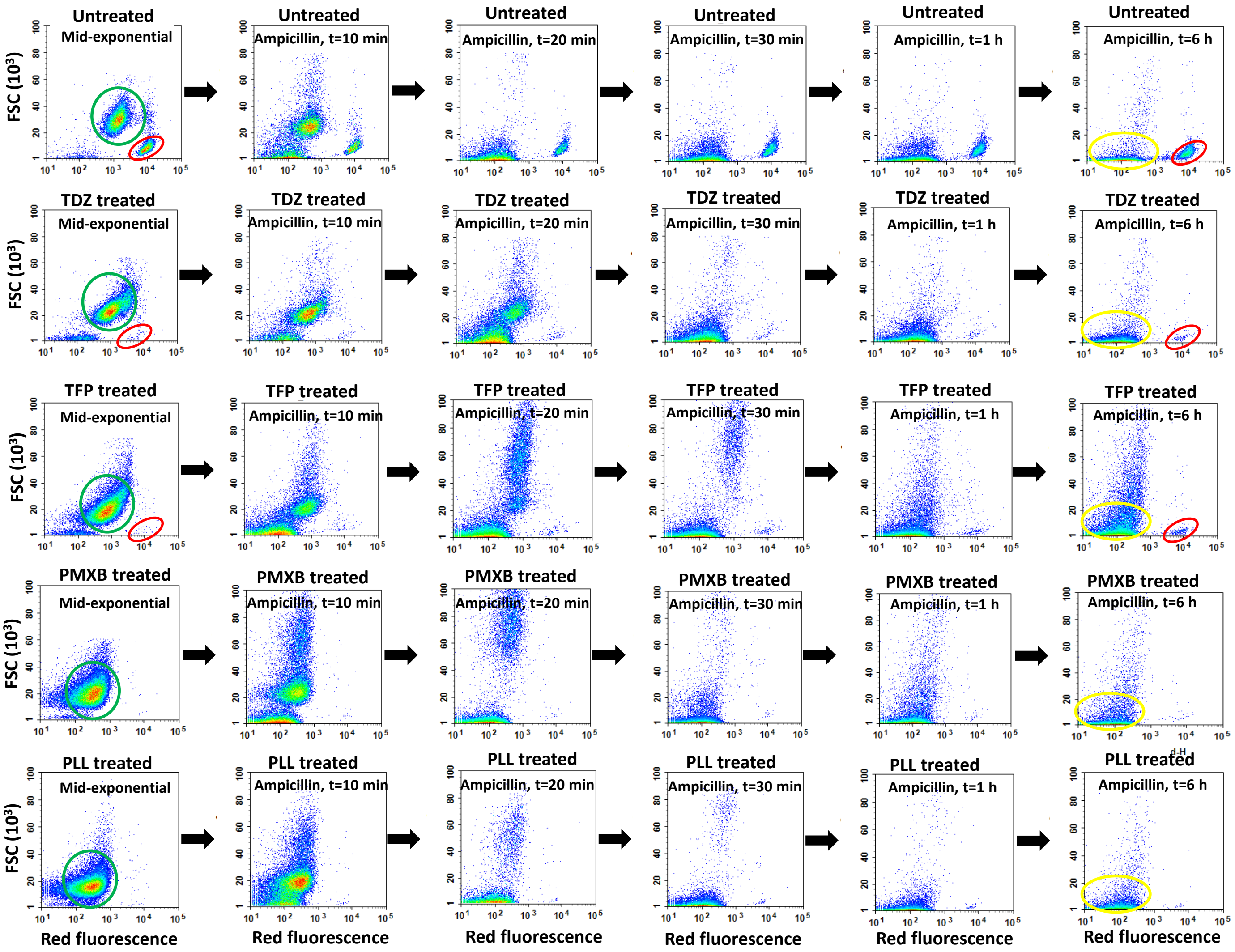

Fig. S9. Effects of chemical treatments on VBNC cell levels. Growing cells (green circle), non-growing intact cells (red circle) and debris (yellow circle) were determined as described in Fig. S3. Note that a representative biological replicate is shown here. All 3 biological replicates consistently resulted in similar trends. Drug concentrations: 0.5 mM Thioridazine (TDZ); 0.5 mM Trifluoperazine (TFP); 0.01 mM Polymyxin B (PMXB); 0.75 mg/ml Poly-L-lysine (PLL).

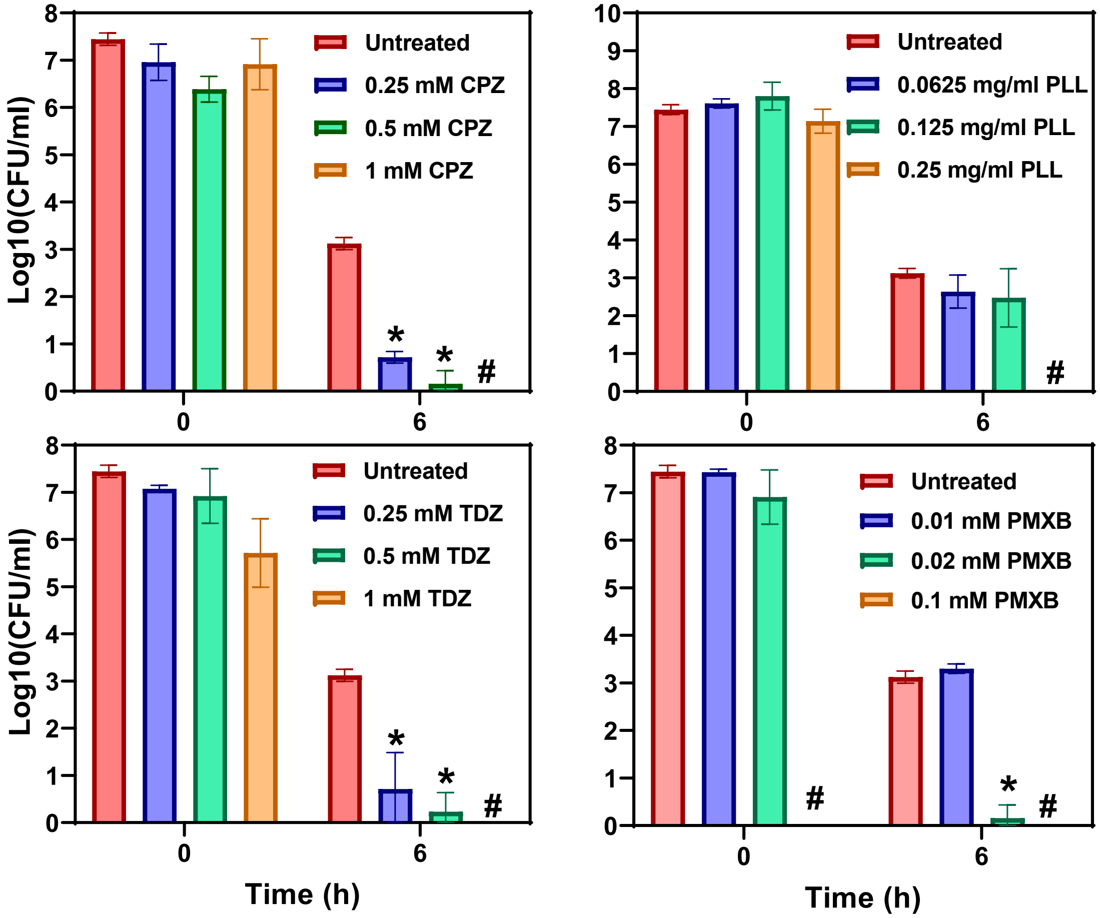

Fig. S10. Persister levels in *P. aeruginosa* cultures treated with the chemical hits. *P. aeruginosa* cells at t=5 h were treated with the selected drugs or left untreated (control); cells in late stationary phase were then washed to remove inhibitors and re-suspended in fresh media with ofloxacin (effective for *P. aeruginosa*) for persister assays. Cells were plated for CFU enumeration before and after the ofloxacin treatments to assess the effects of drugs on *P. aeruginosa* cell viability and persistence, respectively, (N=3).

* Statistical significance between drug-treated and untreated cultures (P<0.05, one-way ANOVA with Dunnett's posttest).

# indicates "under limit of detection".

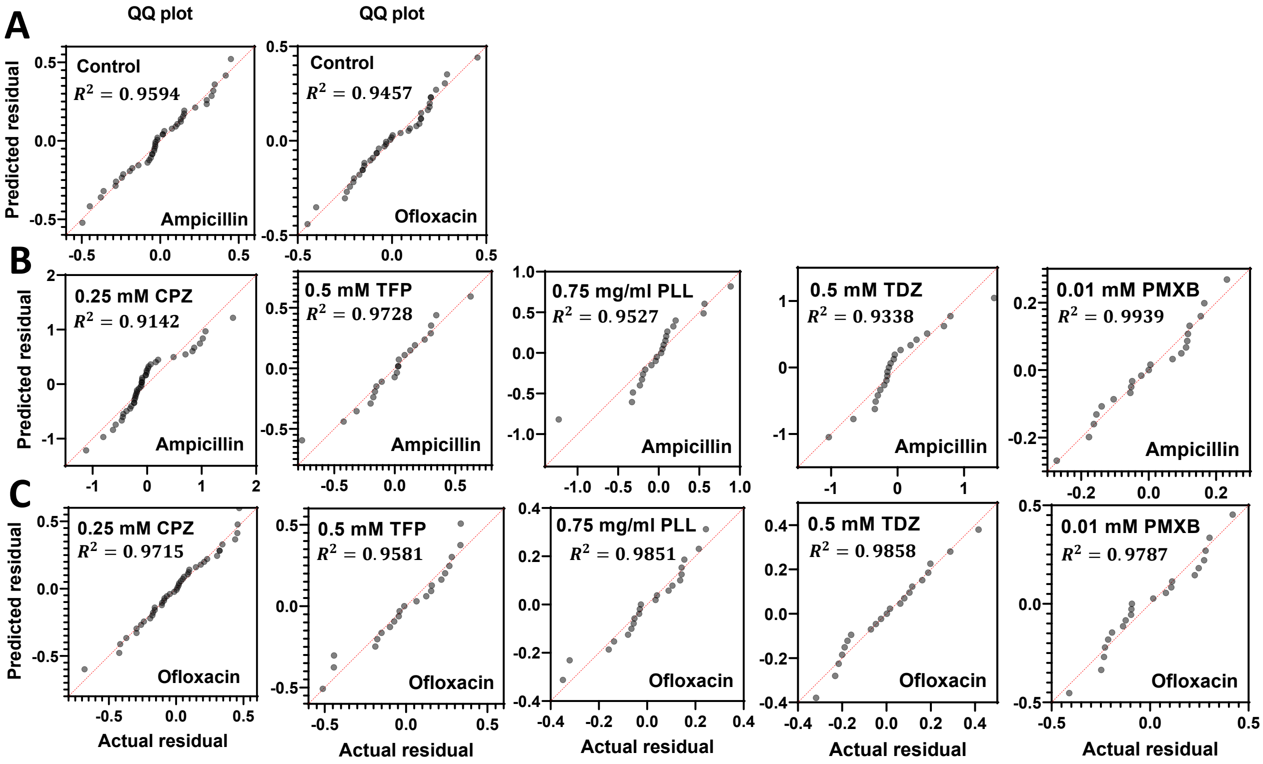

Fig. S11. Quantile-Quantile (QQ) normality plots. (A) QQ plots for the datasets corresponding to ampicillin and ofloxacin kill curves of control groups (no chemical treatment). **(B)** Ampicillin kill curve datasets obtained from cell cultures treated with hit drugs **(C)** Ofloxacin kill curve datasets obtained from cell cultures treated with hit drugs. These figures were generated for each data set by plotting actual residuals vs. predicted residuals sampled from a Gaussian distribution with a built-in function in GraphPad Prism 8.3.0.

Table S1. Bacterial strains used in this study.

Strain	MIC Range (μg/ml)		Source or Reference
	**Ampicillin**	**Ofloxacin**	
E. coli MG1655 wild type	3.125-6.25 (this study)	0.039-0.078 (this study)	(Amato et al., 2013)
E. coli MG1655 + pQE-80L (*ssrA*-tagged *gfp*)	Not detected	Not detected	(Orman and Brynildsen, 2015)
E. coli MG1655 MO strain	Not detected	Not detected	(Orman and Brynildsen, 2013)
P. aeruginosa (PA01)	Not detected	0.3125-0.625 (this study)	(Tam et al., 2005)
**Plasmid**			
pQE-80L (*ssrA*-tagged *gfp*)			(Orman and Brynildsen, 2015)
